# The Natural History Museum Data Portal

**DOI:** 10.1093/database/baz038

**Published:** 2019-04-11

**Authors:** Ben Scott, Ed Baker, Matt Woodburn, Sarah Vincent, Helen Hardy, Vincent S Smith

**Affiliations:** Department of Life Sciences, Natural History Museum, London, UK

## Abstract

The Natural History Museum, London (NHM), generates and holds some of the largest global data sets relating to the biological and geological diversity of the natural world. A majority of these data were, until 2015, not widely accessible, and, even when published, were typically hard to find, poorly documented and in formats that impede discovery and integration. To better serve the bespoke needs of user communities outside and within the NHM, a dedicated data portal was developed to surface these data sets and provide a sustainable platform to encourage their citation and reuse. This paper describes the technical development of the data portal, from its inception to beta launch in December 2015, its first 2 years of operation, and future plans for the project. It outlines the development principles adopted for this prototypical project, which subsequently informed new digital project management methodologies at the NHM. The process of developing the data portal acted as a driver to implement policies necessary to encourage a culture of data sharing at the NHM.

## Introduction

Natural Science collections and the information they contain regarding our knowledge of the natural world and its complexity continue to be digitized at a staggering rate (1–3) and are accompanied by an increase in data that are `born digital’ (4). While we are fortunate to have in our hands an increasing number of tools to assist us with observing, recording and measuring these complex systems, the practice of sharing the wealth of data generated by these activities through publication remains similar to those adopted by naturalists centuries earlier (4). Natural science collections underpin much of this research, and the Natural History Museum, London (NHM), contains one of the largest (circa 80 million objects) including preserved biological and geological specimens collected worldwide over >300 years. In addition to taxonomic and systematic studies, recent advances in digital and genomic technologies are transforming the scientific impact of natural science collections, with huge new potential for addressing societal challenges ranging from biodiversity loss and food security to climate change and neglected and emerging tropical diseases. These unique data are arguably the foundation for meeting the most important challenge humans face over the next 30 years—mapping a sustainable future for ourselves and the natural systems on which we depend (5).

Like many similar institutions, the NHM is undertaking a large-scale program of digitizing its collections (6). The museum's 300+ research staff also generates a large number of research data sets, frequently published as `grey literature’ in the supplementary materials of journal articles, or that remain unpublished after analysis.

These data come in many forms, from digital surrogates of specimens to records of biological interactions and multimedia files associated with specimens from recordings of songs (7) to 3D scans (8).

To expose these data, the NHM commissioned a project to develop a sustainable and publicly accessible web repository with the aim of providing consolidated access to the NHM's collections and research data sets. This project needed to serve as a platform to visualize and explore collections data held in our institutional Collections and Digital Asset Management Systems (CMS and DAMS, respectively), as well as to allow museum scientists to publish data according to open standards (9).

## The Data Portal

The primary purpose of the data portal is to provide a central access point to data produced by the NHM and strategically important partners engaged with NHM research. These cover a diverse range of projects relating to Earth and Life Science Collections, including UK regional museums, universities and international scientific organizations. In all cases, the only precondition is a connection to the museum's scientific work (e.g. the Mark My Bird data set discussed later). Key objectives were to deliver an open access platform to specimen-related data; provide a clean and intuitive interface for exploring and downloading these data including geospatial browsing; provide globally unique and persistent record and data set level identifiers to facilitate scholarly citation, (DataCite DOIs); support user-contributed data sets; provide an application programming interface (API) to access, query and download data according to internationally recognized data standards; and allow major third-party domain aggregators, including the Global Biodiversity Information Facility (GBIF), to harvest collection records from the portal.

### Development principles

At the outset, the project defined a set of development principles, which informed all subsequent decision making and development choices. This process was inspired by the seven original design principles developed by the UK Government Digital Service around the themes of openness and consistency (https://www.gov.uk/guidance/government-design-principles). These principles were adapted by the NHM Informatics Group, which has overarching responsibility for delivering the project, and agreed by the Data Portal Project Board, which has the responsibility to ensure the project is executed in a timely fashion and meets the diverse needs of the Museum's Science and Public Engagement Groups.

- **Do not reinvent the wheel.** Use existing, established technologies wherever feasible, reducing the amount of code that the NHM will need to maintain and allowing us to leverage the existing, active development communities for third-party products.
- **Open by default.** The exposed data sets will be released under open (usually Creative Commons Zero (CC0) or Attribution) licenses and only closed by exception. The platform adopted for the project should be open source and our contributions released under an open-source license.
- **Standards compliant.** Specimen collection data will be mapped to the Darwin Core (DwC) standard and made accessible through DwC Archives (DwCAs) (10), which are internationally recognized within the biodiversity science community. Related data and metadata will map to related international standards including those necessary to meet the requirements of the DataCite organization necessary for us to issue DataCite Digital Object Identifiers.
- **Eat your own dog food** (11). The same public-facing API will be used to construct all functionality of the site to ensure that the API is and always remains production ready. The data portal is a consumer of its own API, so any impairment of the API service will impact the platform before external developers. The process of creating the downloadable data archives is powered by the API, ensuring the API is capable of handling large data requests at scale. In addition, the authors of this paper have used the NHM Data Portal to deposit their own research data sets (e.g. the NHM Sound Collection data sets discussed in `Contributed Data sets’ below).
- **Optimized for data discovery.** User interfaces are intended for researchers' data to be discovered and downloaded. A deeper analysis of the data is better performed in tools designed for the task. Surfacing content for wider public engagement—for example, prettier visualizations embedded on other websites—should be powered by the API.
- **Prototypical with light-touch management.** The data portal was prototypical, to quickly innovate new ideas requiring agile, light-touch management; a preference to release quickly even if it broke things; and a small cell of developers, physically located alongside members of the museum's science staff to ensure stakeholder needs are met.

These principles have been adopted by the NHM software developer community in subsequent projects to streamline innovation while mitigating the risk of feature creep that may result from multiple stakeholders.

**Table 1:** Comparison of open source data portal platforms

Name	Version tested	Technology	Author	Website	Example sites	Technology stack	Documentation	Functionality/extensibility	Popularity and sustainability
CKAN	1.8.1/2.0 Alpha	Python, Pylons and PostgreSQL	Open Knowledge Foundation	ckan.org	data.gov.uk	Extensive experience of Python and PostgreSQL within Informatics Group developers. Pylons framework has been superseded by Pyramid.	Good	Modular and extensible—plenty of examples of open-source plug-ins to build upon. Has a module to integrate with Drupal/Scratchpads. Flexible data storage, we can change to cloud storage systems. Provides support for any type of data set.	Very popular; is becoming the de facto standard for data portals, little risk of obsolescence. Has EU backing and used to build data.gov.uk. OKF employs a team of developers to support it.
						CKAN Version 2 is near Beta now, so we would need to build against the new, less well-documented version.			
Open Data Catalog	1.0	Django, Python and PostgreSQL	Azavea	github.com/azavea/Open-Data-Catalog	opendataphilly.org/	Django is an extremely active and popular python framework. GIS built in.	None	Based on Django, so easily customizable. Supports all types of data sets.	Open Data Catalog does not seem to be widely used and is not even included on the software company's product pages: http://www.azavea.com/products/
Open Government Platform (Version: Alpha)	Alpha	Drupal, PHP, MySQL	Indian and US government	opengovplatform.org(archive)	data.gov	Building on top of Drupal—fits well with museum/scratchpads.	Very limited	Very customizable with Drupal. Supports all types of data sets.	Popularity waning? US moving to CKAN anyway: http://blog.okfn.org/2013/02/01/us-data-gov-ckan/

						Still Drupal version 6 though, and MySQL only; limited geospatial capabilities.		

						It’s the open source version of the US’s Data.gov platform, which works well.		
Customized IPT	1.0	GBIF IPT, Java	Canadensys	github.com/Canadensys	canadensys.net	Built upon the GBIF IPT—much simpler GBIF ingestion.	Good	Quick win: does have all the functionality we need for the collections data set on the data portal. However, it is very much customized for the needs of Canadensys—extending it to allow depositing data sets/cloud storage will be a lot of work.	Not future proof: looks to have just one (judging from the git repo logs) developer maintaining it who was not the original creator. Not a common approach—seems to be the only portal built with a customized IPT
						Java—limited experience within Informatics Group developers.			

(Continued)

**Table 1:** Continued

Name	Version tested	Technology	Author	Website	Example sites	Technology stack	Documentation	Functionality/extensibility	Popularity and sustainability
Nodes Portal Toolkit	NPT 1.0	Drupal, PHP, MySQL	GBIF	nodesportaltoolkit.blogspot.com	nptstartup.gbif.org(archive)	Drupal (currently version 6; 7 in development)	Limited	Built with Drupal so easily extensible. Designed for biodiversity data sets—extending to support any type of data set will require extensive customization.	Appears to have just one developer performing the upgrade from Drupal 6 to Drupal 7.
DataVerse	3.5.0	Java, PostgreSQL	IQSS Harvard Library Harvard University Information Technology Harvard-Smithsonian CfA	thedata.org	dataverse.nl/	Java—limited knowledge with Informatics Group	Good	More focused on publication data—most sites are universities and libraries. UI and design is awful. Aimed at researchers.	Under active development, with analytical tools planned for future versions. Limited take-up outside of initial partners. Very few portals are being built with it.
DSpace	1.8.1	Java, PostgreSQL/Oracle	1000s of universities http://www.dspace.org/whos-using-dspace	dspace.org	spiral.imperial.ac.uk/	Java—limited knowledge with Informatics Group	Good	Designed for academic and research libraries/unis as an open access repository. Has a lot of the functionality we need. And more besides. Is probably overkill for what we want to do—collection management tools built in etc., is much more than an open access portal.	Lots of UK institutions using it—Imperial etc.,

### Implementation (CKAN)

The NHM Data Portal is a customized version of Comprehensive Knowledge Archive Network (CKAN; http://ckan.org/), the open-source data portal software developed and managed by the Open Knowledge Foundation. CKAN was chosen in 2013 from a number of different open-source portal options, which were assessed according to technology stack, documentation, functionality/extensibility and popularity/sustainability (summarized in Table 1). A key requirement was the alignment of the platform stack with existing skills and competencies within the NHM developer team, so PHP- and Python-based solutions were preferred to Java. Of those, CKAN was judged to have the most significant traction with major data providers, including governments (http://data.gov and http://data.gov.uk) and universities (12), as well as superior documentation and active developer adoption. The software is open source and all deposited data sets are open by default; its metadata complies with the Data Catalog Vocabulary (DCAT; https://www.w3.org/TR/vocab-dcat/) standard; user interfaces and download mechanisms provide a rich interface to explore data, directly leveraging its own API.

CKAN is a modular system, and the base functionality can be customized and extended with extensions. Many existing data projects (data.gov.uk, publicdata.eu and German Open Government Platform) have open-sourced their modules, so that others can build upon these.

### CKAN data model overview

**Figure 1:**
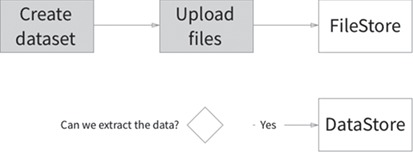
Data set FileStore and DataStore model.

**Figure 2:**
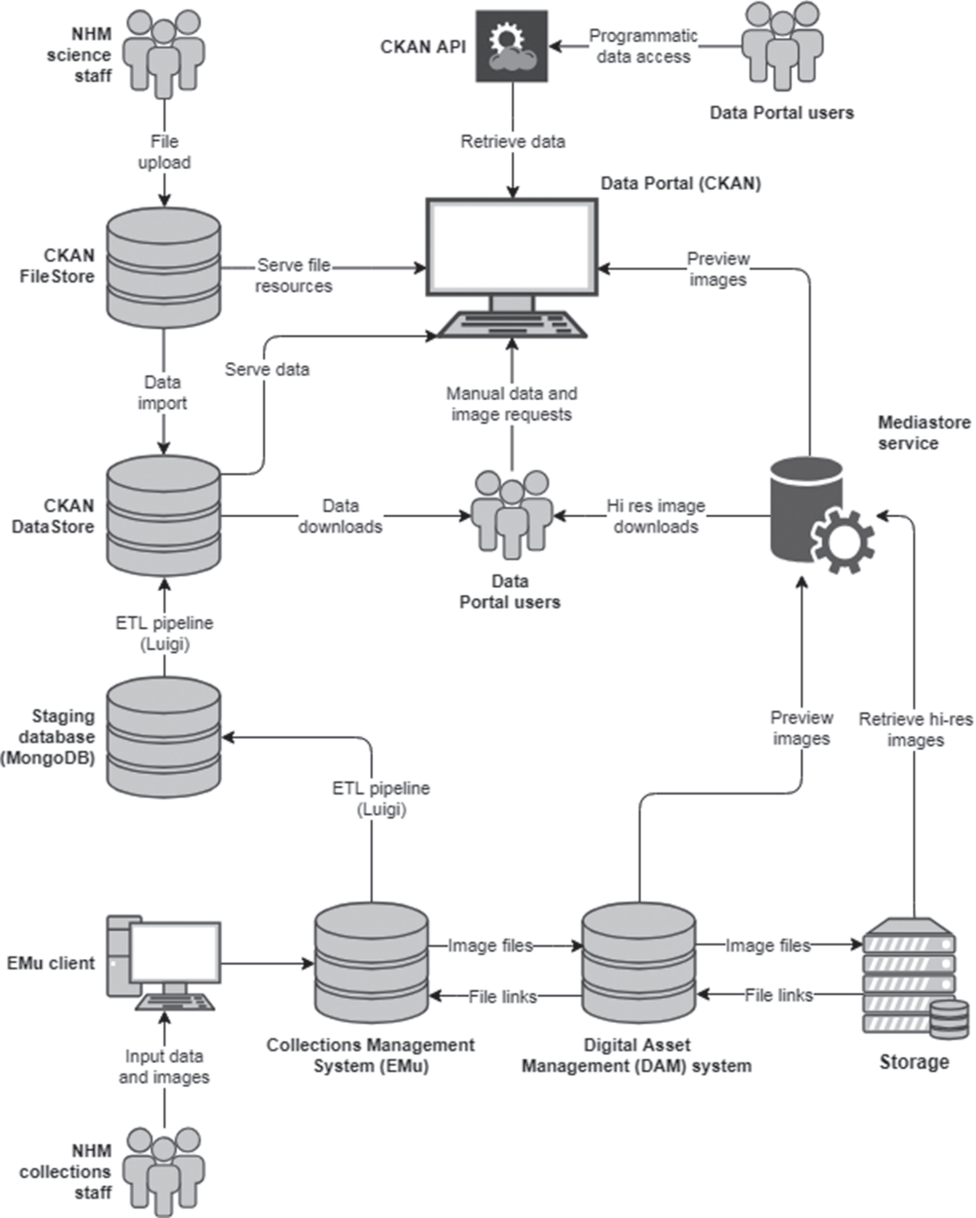
Overview of the technical architecture for publishing collections data and digital media.

Data sets are the central entity in CKAN, acting as `umbrella’ objects holding the metadata and one or more resources (a file, URL or other resource). When a resource is uploaded to CKAN, the file is processed and stored according to its data type. All uploaded files are added to the FileStore, a persistent local or cloud file storage location. The data portal uses network file storage on the NHM network. FileStore objects can be discovered using their data set metadata and only downloaded in their entirety; CKAN is essentially agnostic about their contents. However, if one of these files contains structured information in a known format—for example, an Excel or CSV spreadsheet—a further processing step takes place and the data are extracted and imported into the DataStore (Figure 1). The DataStore provides a database for structured storage of data, in which individual spreadsheet rows can be accessed and queried via the web API. Figure 2 summarizes the CKAN components and their interactions with other key architectural elements involved in collections data and digital media management.

### CKAN interfaces and visualizations

By default, CKAN provides three visualizations for structured data: tabular, graph and map. These views are paged in a way that, while fine for structured data set resources with a limited number of records, is less optimal for larger data sets; only the first 200 records are displayed on the map and graph. If a user searches for a species in our collection, we want to display species distribution across the entire world.

**Figure 3:**
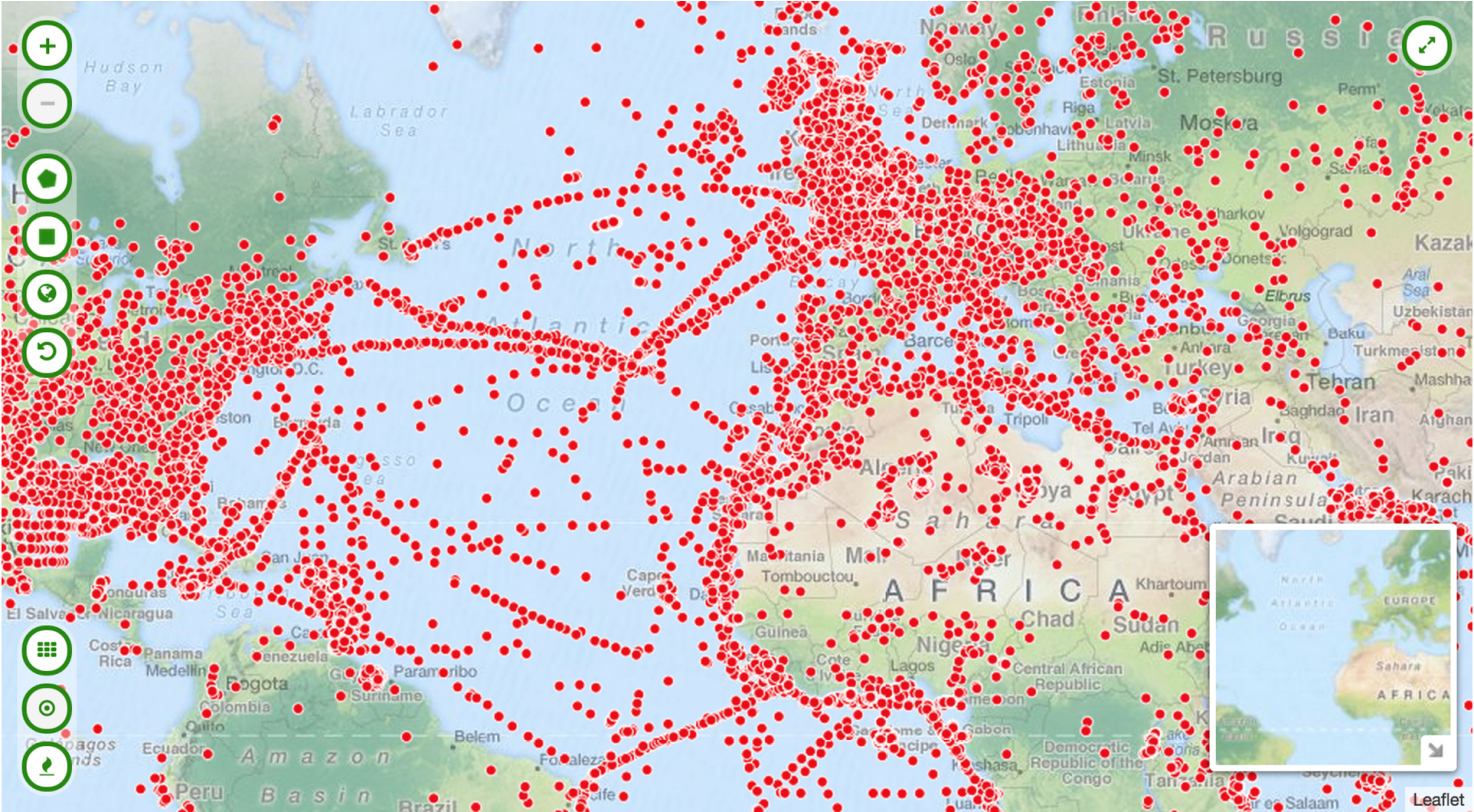
Interactive map visualizing over one million geocoded collection objects.

We developed a view plug-in for CKAN that allows display of millions of data points on an interactive map. In line with our core principle to use existing technologies wherever possible, we adopted the same technologies built and open-sourced by CartoDB for its mapping platform: Windshaft (https://github.com/CartoDB/Windshaft), a node.js library for PostGIS and torque.js, which renders map tiles on the server. Client-side, the maps are kept quite simple; a user can toggle between viewing records as points or density (heat- or choropleth-) maps (Figure 3). This simplicity is by design—the map views provide a mechanism to surface the data; once downloaded in a standard format, a user can choose to perform more complex analyses of the data in one of a number of the geospatial systems designed and optimized for the task.

**Figure 4:**
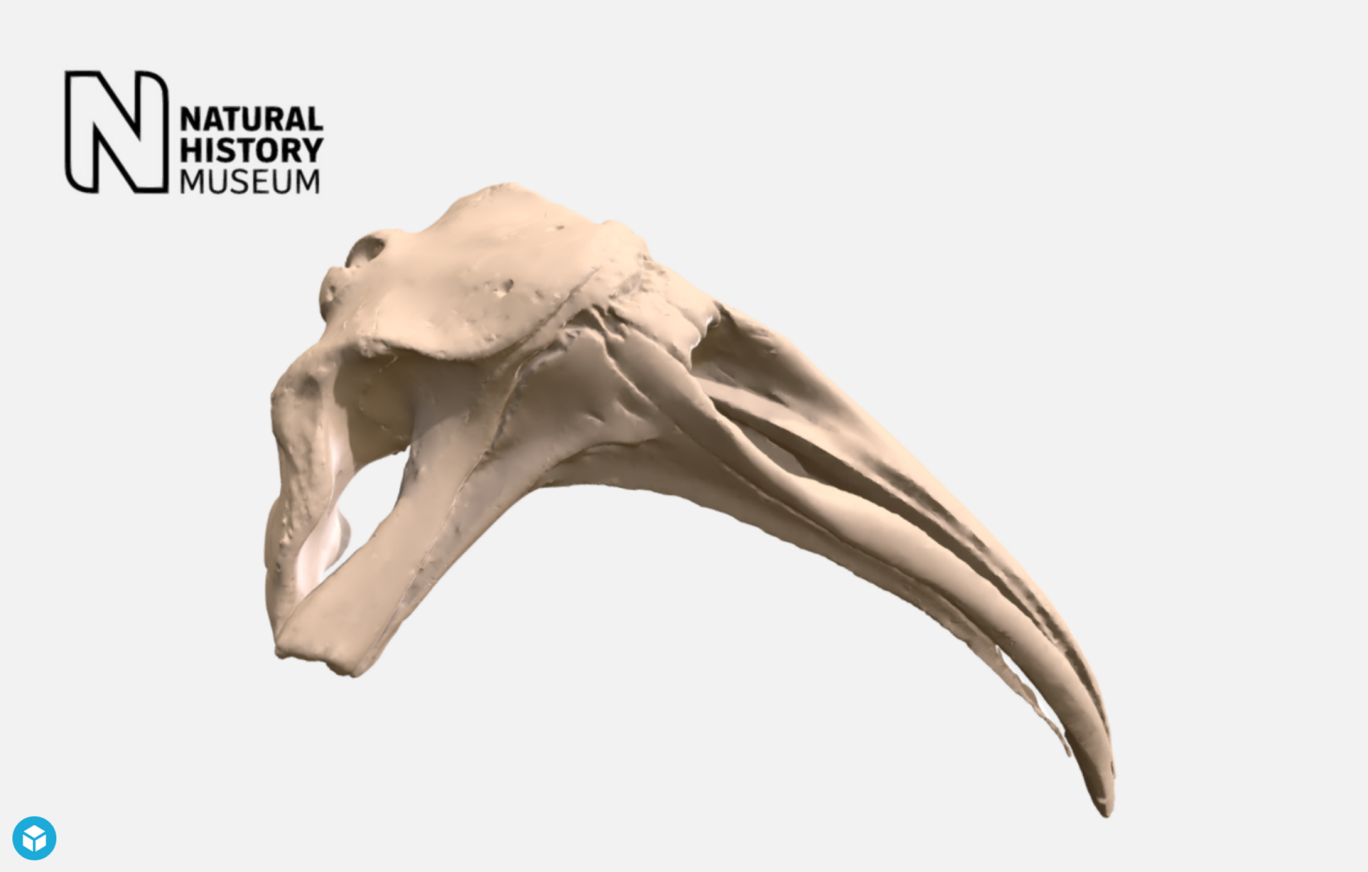
Sketchfab 3D model of southern right whale cranium http://data.nhm.ac.uk/dataset/3d-cetaceanscanning/resource/63a6168b-4594-4998-964e-86b8f7398e9c.

We also provide three other custom view interfaces: a gallery (for displaying grid views of pictures), video (embedding resources from Youtube and Vimeo) and Sketchfab (embedding 3D models; Figure 4).

CKAN does not provide a record level view of each item in a data set. This custom functionality was developed in house and incorporated into the NHM plug-in. This atomic view was extended for the collections data set, augmenting the basic record view with DwC and GBIF views. The DwC view displayed the data grouped by DwC terms; the GBIF view shows the data as interpreted by GBIF, with links to the GBIF taxonomic backbone and occurrence record.

### Accessing data

**Figure 5:**
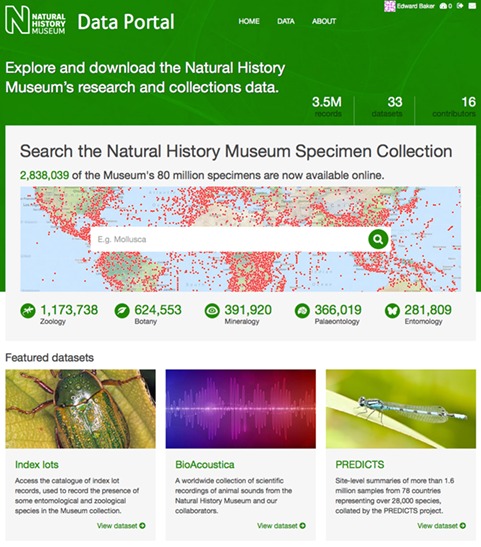
The data portal homepage.

Data from the NHM Data Portal can be accessed in a variety of ways. The primary interface is the website http://data.nhm.ac.uk (Figure 5). Data sets and metadata can be searched using an integrated instance of Apache Solr (http://lucene.apache.org/solr). All data set resources can be downloaded—users are required to enter their email address and the selected data set resources will be packaged and a link emailed to them. If the data set is a structured resource within the DataStore, the user can select the individual rows to include in the data package. A bespoke module was developed to enable packaging of our large collection data sets, transferring the request to an asynchronous backend process.

The data portal provides a powerful RESTful read/write API. All data portal core functionality is available via the API (endpoint available at http://data.nhm.ac.uk/api/3; documentation at https://docs.ckan.org/en/2.8/api/). For example, the GET request http://data.nhm.ac.uk/api/3/action/datastore_search?resource_id=05ff2255-c38a-40c9-b657-4ccb55ab2feb&q=archaeopteryx searches the NHM collection data set for records related to Archaeopteryx. The API has been used in data analytics, custom visualizations (http://naturalhistorymuseum.github.io/specimen-globe/), postgraduate training courses and hackathons (e.g. Open Data Day, http://opendataday.org/; Over The Air, http://overtheair.org) and to provide access to the data portal from the R environment for statistical computing (13).

The data portal is also available as machine-readable Resource Description Framework (RDF; https://www.w3.org/RDF/). Every page on the portal is available as Notation3 (N3), Turtle (TTL), JSON-LD and RDF/XML and can be requested in a machine-readable format by setting the appropriate HTTP request header. Data set and resource metadata are mapped to Dublin Core, vocabulary of interlinked data sets and DCAT. Collection records are mapped to DwC. In addition, by exporting our collection records to GBIF and reloading the resulting GBIF-parsed data set back into the data portal, complete with GBIF's links to its taxonomic backbone, we can provide the collections data set as true linked open data (LOD). For example, an unprocessed data portal DwC record (http://data.nhm.ac.uk/dataset/collection-specimens/resource/05ff2255-c38a-40c9-b657-4ccb55ab2feb/record/3135317) can be represented in RDF triples as shown in Table 2.

The predicate is a string value exported from the EMu collection management database. After reloading the data from GBIF, the same record values can be represented as outbound links to other classifications, including GBIF, Catalogue of Life and Biodiversity Heritage Library (Table 3).

**Table 2:** Linked open data before processing using GBIF

Subject	Object	Predicate
http://data.nhm.ac.uk/object/f4df8e22-15d0-4786-81b1-24bdf049ec5e	https:// dwc.tdwg.org/terms/#scientificName	`*Trisopterus luscus* (Linnaeus, 1758)’
	https:// dwc.tdwg.org/terms/#genus	*`Trisopterus’*

**Table 3:** Linked open data after processing using GBIF

Subject	Object	Predicate
http://data.nhm.ac.uk/object/f4df8e22-15d0-4786-81b1-24bdf049ec5e	https://dwc.tdwg.org/terms/#scientificName	https://www.gbif.org/species/2415916; `*Trisopterus luscus* (Linnaeus, 1758)’
	https://dwc.tdwg.org/terms/#genus	https://www.gbif.org/species/2415905; *`Trisopterus’*

**Table 4:** CKAN packages used by the NHM Data Portal

Package	Description
ckanext-ckanpackager	Provides a user interface to download resources using ckanpackager.
ckanext-contact	Contact form.
ckanext-datasolr	SOLR to index and search data sets (used for specimen collection).
ckanext-dataspatial	Adds geospatial searches within the datastore.
ckanext-dev	Developer and debugger tools.
ckanext-doi	Integration with DataCite to create DOIs.
ckanext-gallery	Data set resource image galleries.
ckanext-gbif	Loads the GBIF data set back into the portal.
ckanext-graph	Server-side graph rendering.
ckanext-ldap	LDAP integration—allow staff to login with their museum account.
ckanext-list	List view of resource records, displaying a subset of fields.
ckanext-map	Geospatial visualization of records.
ckanext-nhm	Main NHM extension, providing theming and generic customizations.
ckanext-sketchfab	Embedding Sketchfab 3D models.
ckanext-statistics	API for accessing data portal metrics.
ckanext-status	Status banner for system alerts.
ckanext-twitter	Twitter integration, for tweeting when data sets are created and updated.
ckanext-userdatasets	Allow users with `member’ role within an organization to create/edit/delete their own data sets.
ckanext-video	Embedded Youtube and Vimeo video players.

This is the first instance of utilizing GBIF's aggregation and taxonomic name resolution service to automatically produce a LOD collection data set. As a result, the NHM remains one of the few institutional data portals to achieve a 5-star rating in Tim Berners-Lee's Open Data deployment schema (https://5stardata.info/en/). Although the LOD data set has not yet (to our knowledge) been widely exploited, there have been some uses, for example as part of the BBC Research and Education Space initiative (https://bbcarchdev.github.io/res/) to connect public archives and digital collections as a resource for education. Within the biodiversity community, upcoming collaborations such as the DiSSCo initiative (https://dissco.eu/) are also beginning to focus more attention on semantic linkage and enrichment of collections data.

### Code repositories

Code repositories (GitHub) used by the data portal are listed in Table 4. As the code is available under a variety of open licenses (e.g. MIT or GPL-3.0), we cannot track instances of use, except when issues are formally raised in GitHub by other developers. From this, we are aware of several instances where our extensions to CKAN have been exploited by others, one of the most popular being the NHM's LDAP module that supports user authentication. In addition, some NHM extensions have been adopted into the core CKAN codebase, such as several relating to `chained actions’. However, we are not aware of any instances where peer institutions have adopted the entirety of the NHM Data Portal, despite receiving expressions of interest from several natural science collections. We remain open to this possibility, and through new collaborations such as the recent DiSSCo initiative (https://dissco.eu/) that is working to bring together the digital infrastructure for European natural science collections.

## Data Sets

### Collections

**Figure 6:**
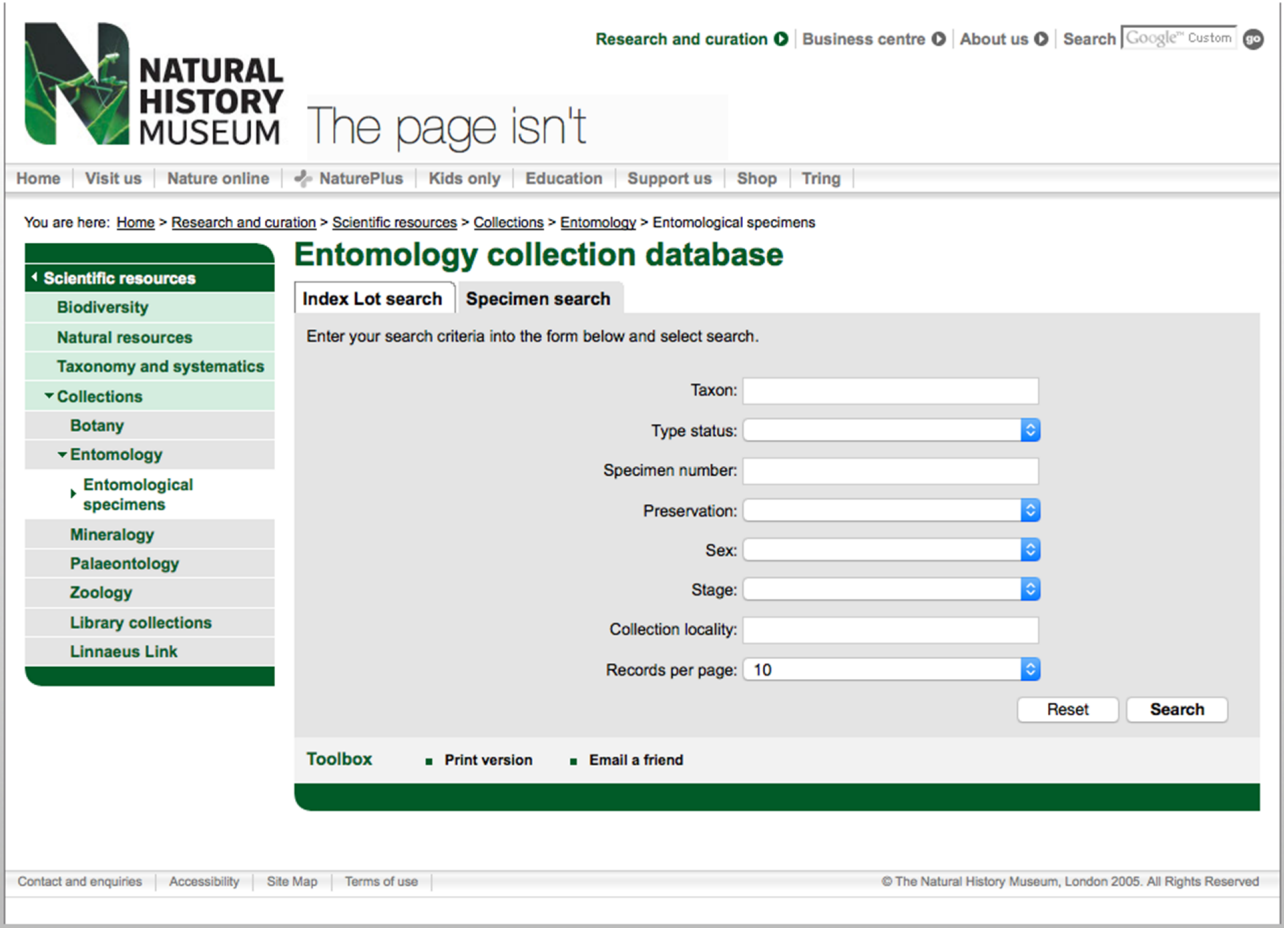
The old web search interface to the Entomology collections of the NHM.

The NHM currently uses Axiell's `EMu’ as its CMS. Prior to the newly developed data portal, a subset of this database, the web-safe version, was exposed via the NHM website with a custom search interface (e.g. Figure 6). The web-safe version has a number of records removed for collections security, species conservation and where data is under an embargo e.g. during active research. While this existing interface did allow researchers to surface information, it did not provide access to the data themselves and was superseded by the portal with its richer web and data interfaces. While no data exists on the usage of the original web-safe subset of the collection data, the absence of any feedback from prior users, coupled with the positive feedback received when the data portal launched, suggests that this original version was not missed.

**Figure 7:**
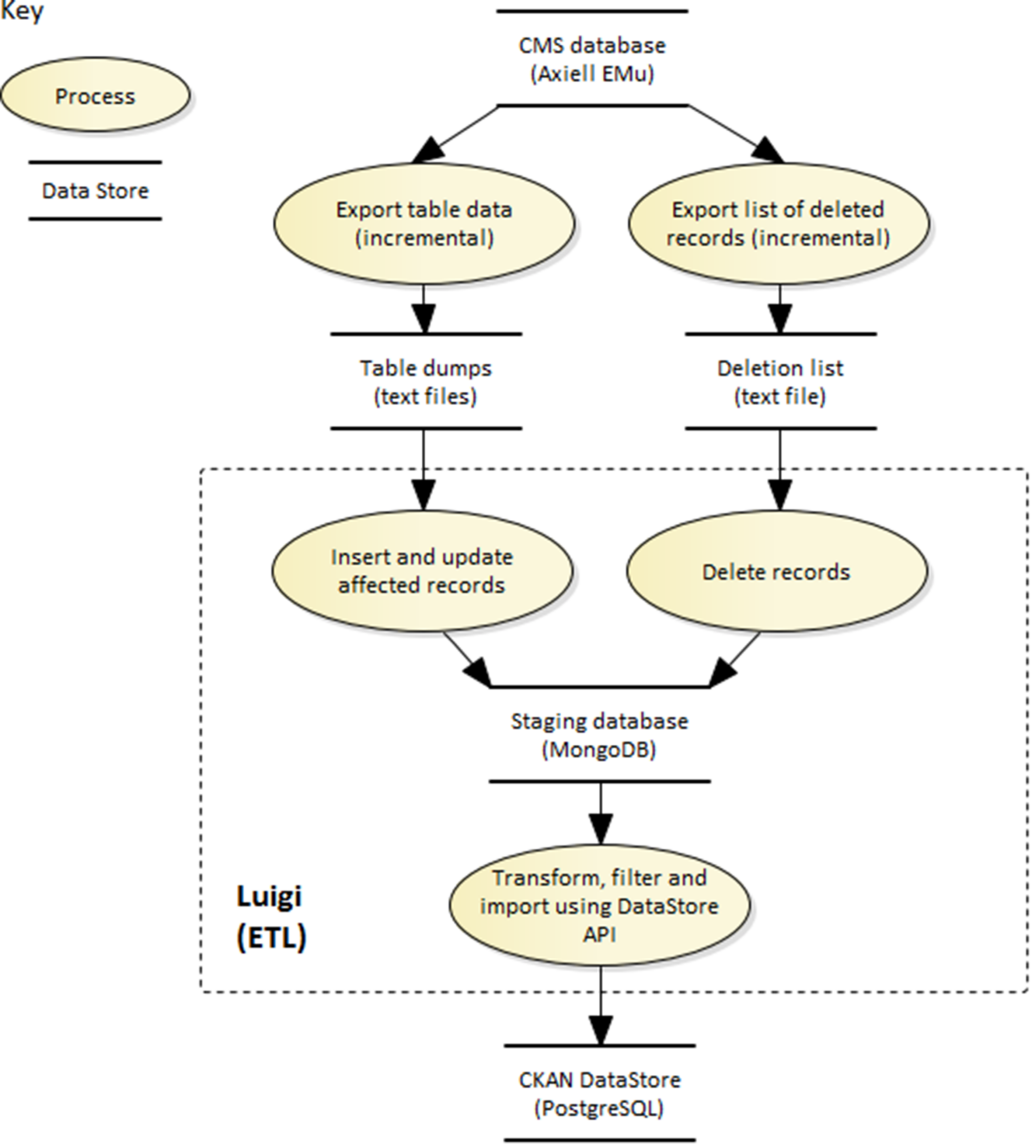
The Luigi ETL pipeline for loading KE EMu collection records into the data portal.

The Data Portal database of NHM specimens currently exceeds 4 million records, and with over 80 million objects in the collection, coupled with an active digitization program, the data portal is designed to scale as the number of records grows.

#### Mapping and ingest of EMu data

To make the collections data available on the portal, we needed to retrieve the records from the EMu CMS and transfer them to the portal. This was a far from simple task. There was no functioning EMu API that could be used to access these records at scale. Instead, the data had to be exported from EMu and imported into the data portal. To ensure the collection records on the data portal would not get stale, the EMu records would be exported and ingested at frequent intervals.

At launch, these were produced at weekly intervals, but since 2016 have been produced 5 days a week. This has reduced the number of records included in each export (Table 5), shortened the publication pipeline and significantly improved the currency of the collections data.

**Table 5:** EMU exports and record counts per annum (High total record count for 15/16/17 caused by repeated full data reload events.)

Calendar year		Total records exported	Mean records per export
2015	31	4 302 239	138 781
2016	52	6 983 021	134 288
2017	163	6 030 596	36 997
2018	257	367 625^*^	1430

The object relational data model in EMu, coupled with the historical migration of data from the NHM's legacy of department-specific (and, in some cases, taxon-specific) databases, has resulted in a heterogeneous set of collection records. These commonly involve over a thousand fields, which include the duplication of field concepts by the different collection departments and as a result these records are often sparsely populated. As a result, the information from EMu cannot simply be regurgitated onto the public-facing portal and requires substantial mapping and reformatting into records conforming to DwC (9).

The extract, transform and load (ETL) pipeline built to transfer data from EMu to the data portal is orchestrated by Luigi (https://github.com/spotify/luigi), an open-source framework built by Spotify for managing complex pipelines of batch jobs. Luigi handles dependency resolution, workflow management, visualization, failure handling and command line integration. It was chosen above other batch orchestration toolkits for its ease of use and flexibility; tasks are programmed within python, not defined in configuration files, and can be integrated with any data source. Each task in a pipeline is an independent entity. If a task fails, it will notify and potentially block subsequent dependent tasks. For the data portal pipeline, one task retrieves and reads the EMu export file. The next imports the data into MongoDB (its schema-less JSON document storage provides an easy staging area for EMu object-oriented database records). The data is then queried from MongoDB and transformed into DwC. The final task writes the DwC data to the data portal via the DataStore write API. An overview of the architecture involved in this process can be found in Figure 7.

This process again reflects our ongoing commitment to the core development principles outlined at the project's inauguration; we leverage existing technology (Luigi) to construct the pipeline rather than write our own; the data portal's own API is used to write the data into the system.

#### Data standards

To ensure the discoverability and utility of data sets released on the portal, as well as to facilitate interoperability with other systems, data standards have been adopted at many levels of the system. Data sets and their resources conform to Dublin Core and DCAT metadata standards, with additional elements from HYDRA (http://www.hydra-cg.com/spec/latest/core/) for describing data set index results and INSPIRE (https://inspire.ec.europa.eu/) for data sets including a geospatial component. These metadata fields additionally conform to the DataCite Metadata Schema (https://schema.datacite.org/), allowing the data portal to mint a DataCite DOI for every public data set. At present, the DOIs resolve to the most recent version of the data set as persistent historical versions of the data are not supported. An upcoming release of the data portal will add this functionality, enabling minting of persistent DOIs for historical versions and subsets of the collections data sets.

Collections records can be downloaded in the standard DwCA format [a single zip archive of files defined by DwC (10)], which is widely used for data sharing in biodiversity informatics (14). For user-contributed data set resources, conforming to a standard is encouraged but not prescribed. This remains at the discretion of the depositee, to maximize the release of open data through the portal. In many cases, museum scientists are best placed to understand the utility of their data sets within their peer scientific communities and align with data standards relevant to their research domain. In this respect, we seek to make data curation a self-regulating exercise, so long as minimum metadata standards are adhered to. The mandatory minimum metadata fields are the following:

- Data set title
- Abstract
- Data set category
- Author

#### Mediastore integration

The NHM uses a DAM system to store digital assets, including images uploaded to EMu. Collection images are displayed on the portal via the DAM API, which returns media assets at a suitable web resolution. The data portal also provides an interface to request the original image. The NHM archives digital assets long term on magnetic tape. The requestor is required to enter their email address and will subsequently receive an email with a link to the original media file, once retrieved from tape and made available on a web-accessible staging area.

#### DQIs

**Figure 8:**
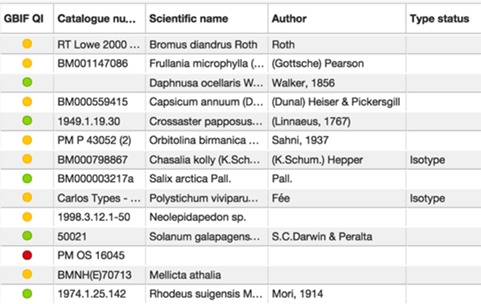
View of NHM specimens on the NHM Data Portal showing DQIs from GBIF (green, no known errors; orange, minor errors; red, major errors).

GBIF has developed a number of tools to highlight likely errors in the data sets it processes. The NHM Data Portal contributes the collections data set to GBIF, but also harvests the data quality indicators (DQIs) from GBIF so they can be displayed alongside the collections data within the portal. The DQIs are provided in a traffic-light format (Figure 8; green: no known errors, orange: minor errors, red: major errors), alongside textual descriptions of any problems. These indicators allow curators to find and correct errors within the underlying EMu collections database and external users to gain a quick overview of the likely quality of the data they wish to use. At present, they only extend to life science collections (extant species), due to the absence of services supporting paleontological and mineralogical species.

**Table 6:** Example of metadata for the BioAcoustica contributed dataset (20)

**Field**	**Description**	**Example**
Title	The name of the data set	BioAcoustica
Abstract	Short description of the data set	A worldwide collection of scientific recordings of animal sounds from the NHM and our collaborators
Keywords	Keywords	Bioacoustics, biodiversity, sound, taxonomy
Data set category	Broad theme of the data set	Research
License	How is the content licensed?	License not specified (BioAcoustica has a fine-grained system of licensing individual items of content)
Visibility	Public or private	Public

The portal also enables end users to contact relevant museum curators by email to report errors in the underlying data sets. This allows errors to be identified, reported and fixed using a crowd-sourced approach, ensuring the quality of the NHM's collections data set is constantly improved through gradual refinement.

#### Data sharing

The collections data set contains information about specimens in the NHM collection. These data are shared with regional and global data aggregators who combine the NHM data with data from other institutions around the world. In this way, the NHM Data Portal allows the museum to contribute automatically to a global ecosystem of aggregators and users. At present, the collections data set is shared with the GBIF, VertNet (15), iDigBio and Centro de Referência em Informação Ambiental (16).

#### Stable URIs

The data portal assigns a unique and permanent Uniform Resource Identifier (URI) to each specimen. This follows LOD principles (see www.w3.org/tr/ld-bp) by including a redirection facility to human- and machine-readable representations of the specimen (17). The importance of stable and persistent identifiers has been discussed widely by the biodiversity informatics community [e.g. (18)] and will, in the longer term, allow for much larger initiatives based on semantic technologies to be developed (19).

## Contributed Data Sets

The front page (Figure 5) of the NHM Data Portal highlights featured, high-impact, data sets from our collections and research staff. All museum staff is able to upload data sets in one of the following categories: Citizen Science, Collections, Corporate, Library and Archives; Public engagement; and Research.

### DataCite DOIs

DataCite (http://datacite.org/) DOIs are assigned to all published data sets on the portal. In compliance with the DataCite Metadata Schema (https://schema.datacite.org/), the portal collects metadata associated with each data set. The data portal DOIs are not currently versioned to reflect data set updates, but future iterations will implement this.

#### Metadata

The metadata fields for each data set are provided in Table 6.

### Licensing

The Museum's Digital Collections Programme has created a licensing framework that supports the open licensing of museum data sets, including those that are made available through the NHM Data Portal. In broad terms, this allows for releasing of the collections and research data sets (with associated metadata) under the permissive CC0 waiver. Digital media assets are released under the Creative Commons Attribution (CC-BY) license. Exceptions to these guidelines are made in a small number of cases for pragmatic reasons.

**Figure 9:**
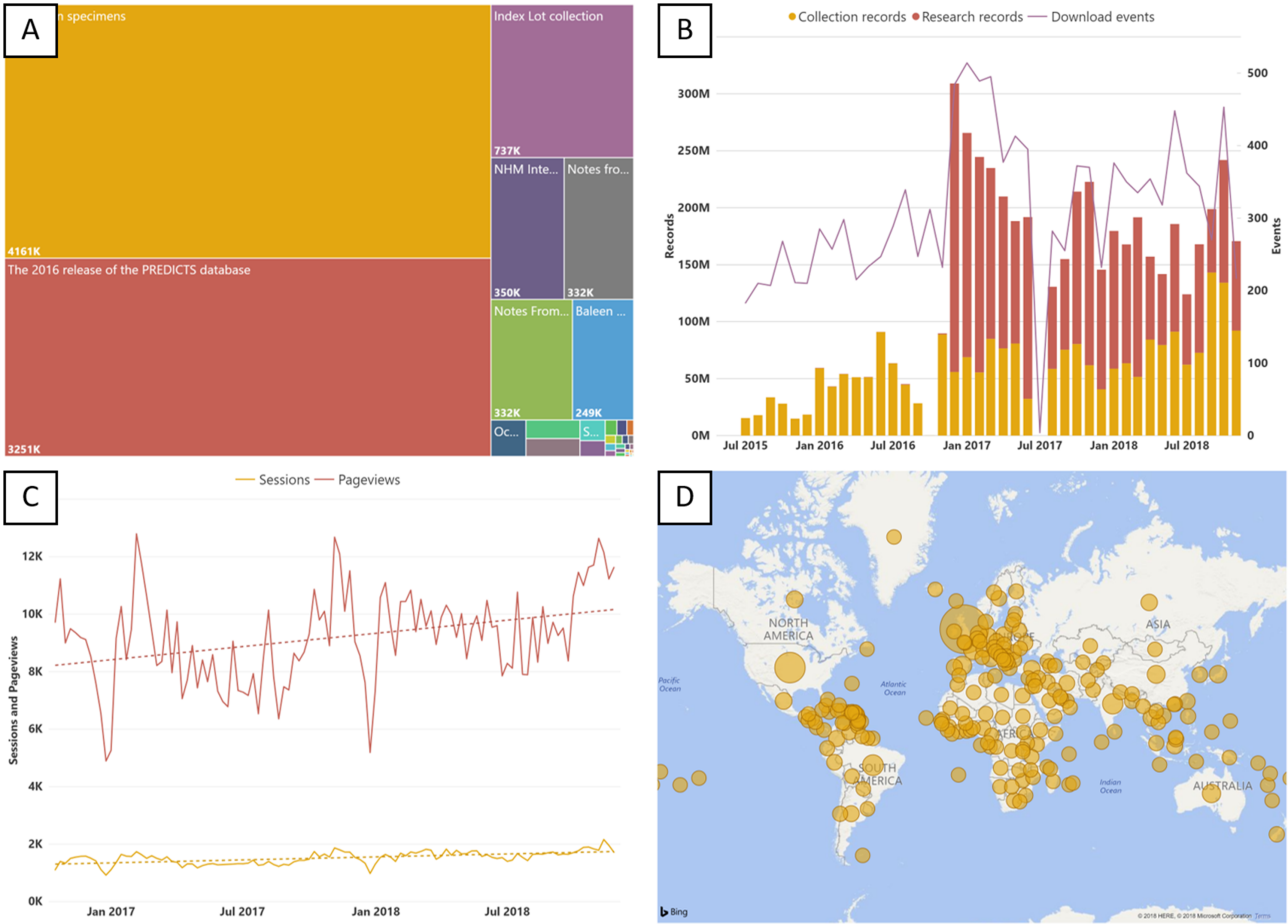
(A) Treemap of data sets hosted on the NHM Data Portal, size reflects the number of records. (B) Records downloaded from the NHM Data Portal each month. (C) NHM Data Portal Web traffic (page views and sessions). (D) Country of origin for users of the NHM Data Portal since launch.%”.

### Highlights of contributed data sets

The NHM Data Portal has already been used as a repository for a number of data sets that underpin core NHM research on understanding the natural world. Museum staff has used the portal to create standalone data sets, data sets that are supported by a data paper and data sets that support a traditional publication [e.g. (21) and (22)]. These data sets underpin work on museum-type specimens (23–24), phylogenetics, bibliographies and species checklists. The data sets cover a broad range of scientific disciplines including botany, entomology, zoology and mineralogy. In addition, the portal contains data sets relevant to several of the NHM's major biodiversity informatics projects including the UK Species Inventory, PREDICTS (25–26) and the Notes from Nature crowd sourcing project (27). The BioAcoustica program (7) has used the portal as repository for its metadata (20), to publish new data including historical recorded talks from the NHM Sound Collection (28–29), and 3D models of the burrows of mole crickets (30).

The portal also hosts a number of more unusual data sets that highlight some of the museum's innovative research programs, including building instructions for a Lego insect manipulator (31–32) and printed circuit board designs for the NightLife aquatic insect trap (33–34). An example of external use of the data portal is the Mark my Bird data set (35), which includes 3D scans of bird bills from the ornithology collection used in a recent publication (36).

## Usage Statistics

The NHM uses Microsoft Business Intelligence to monitor growth and exploitation of data published through the data portal (Figure 9). An example of one of the Museum's Published Dashboards can be accessed at data.nhm.ac.uk/metrics.

These dashboards feed into the museum's internal reporting structures and help build the case for increasing the proportion of our digitized collection.

### Software development and culture change roadmap

The data portal was a prototypical project, intended to launch quickly, if imperfectly. This was the first time the museum had embraced such an approach for a public-facing production website, and the project's light-touch management and small, dedicated team of developers proved remarkably successful; the first beta release of the data portal was built in less than a year, with development starting in January 2014, launched as a closed private beta (NHM staff) in June 2014, with a full publicly accessible beta being launched in December 2014. December 2015 saw the full initial launch of the first phase of data portal development.

In addition to this new approach to development and the corresponding implementation of open source and open standards set out above, the data portal, alongside the museum's program of digitization, catalyzed wider cultural change. In particular, it influenced the museum to adopt an open by default policy to collections data and to determine a managed process for the limited exceptions to this. In most cases—for example where data is embargoed due to ongoing research—exceptions are time limited, with processes to ensure eventual data release. In March 2017, the museum endorsed the Science International Accord on Open Data in a Big Data World, including key principles of open data for open science, and continues to engage for instance in International Open Data Day on social media. As shown in the usage data above, a high proportion of onward use and citation of the museum's digital data is through aggregation, showing the power of sharing data openly across global collections, and of modeling it against other data sources such as climate and population. The further software developments below aim to build on this demonstration of impact and use.

Phase 2 (June 2015 to December 2017) of portal development focused on consolidation of the system, moving from prototype to recognition and use as a key and lasting museum platform: implementing a DevOps-based server architecture, migrating systems to those better supported by NHM technical infrastructure, better documentation, improved reporting of usage metrics, and an improved ETL process. Phase 3 commenced at the start of the 2018 and has been focusing on improving the user design and experience, along with better integration with external systems. Following user interviews and surveys, the data portal is currently being redesigned with a focus on improving usability, particularly around the search interfaces. A unified search will allow users to search across all data sets, resources and DataStore records. In the current system, records are siloed within their respective resources. To improve citability, DataCite DOIs will be minted for each data download request. The data portal will also integrate ORCIDs for data set authors and contributors.

## Notes

**Database URL:** data.nhm.ac.uk
